# Association of Prematurity and Low Birth Weight with Gestational Exposure to PM_2.5_ and PM_10_ Particulate Matter in Chileans Newborns

**DOI:** 10.3390/ijerph19106133

**Published:** 2022-05-18

**Authors:** Alejandra Rodríguez-Fernández, Natalia Ramos-Castillo, Marcela Ruiz-De la Fuente, Julio Parra-Flores, Eduard Maury-Sintjago

**Affiliations:** 1 Department of Nutrition and Public Health, Universidad del Bío-Bío, Chillán 3780000, Chile; alrodriguez@ubiobio.cl (A.R.-F.); marcelaruiz@ubiobio.cl (M.R.-D.l.F.); juparra@ubiobio.cl (J.P.-F.); 2 GABO, Grupo de Investigación en Auxología, Bioantropología y Ontogenia, FACSA, Universidad del Bío-Bío, Chillán 3780000, Chile; 3 FACSA, Universidad del Bío-Bío, Chillán 3780000, Chile; naramos@ubiobio.cl

**Keywords:** newborn, prematurity, low birth weight, particulate matter, public health, air pollution

## Abstract

Fetal growth can be affected by gestational exposure to air pollution. The aim of the study was to determine the association between prematurity and low birth weight (LBW) with gestational exposure to PM_2.5_ and PM_10_ particulate matter in Chileans newborns. This cross-sectional analytical study included 595,369 newborns. Data were extracted from the live newborn records of the Chilean Ministry of Health. Sex, gestational age, birth weight, and living variables were analyzed. We used the Air Quality Information System of the Chilean Ministry of the Environment to obtain mean PM_2.5_ and PM_10_ emissions. A multivariate logistic regression model was performed with STATA 15.0 software at α < 0.05. Prevalence was 7.4% prematurity and 5.5% LBW. Mean PM_2.5_ and PM_10_ concentrations were 25.5 µg/m^3^ and 55.3 µg/m^3^, respectively. PM_2.5_ was associated with an increased the risk of LBW (OR: 1.031; 95%CI: 1.004–1.059) when exposure occurred in the second trimester, while PM_10_ affected the whole pregnancy. In addition, PM_10_ exposure in any gestational trimester was associated with an increased the risk of prematurity. The PM_10_ particulate matter was associated with both prematurity and LBW in all of the trimesters of exposure. The PM_2.5_ particulate matter was only associated with LBW when exposure occurred in the second gestational trimester.

## 1. Introduction

In recent years, the prevalence of preterm newborns (PN) has increased in western countries. The multicausal origin of prematurity is mainly associated with maternal, fetal, socio-environmental, and other factors [1,2,3]. Prematurity is a postnatal condition that can produce sequels in varying degrees, and it is considered the main cause of death and disability of the newborn [4].

Fetal growth and development can be affected by changes in the morphology and placenta resulting from gestational exposure to air and environmental pollution. The main action mechanisms of air pollutants involve systemic inflammation processes, antiangiogenic factors, and oxidative stress [5]. Particulate matter is deposited in the lungs and heart increasing the risk of free radical peroxidation damage, imbalance of intracellular calcium homeostasis, increased proinflammatory cytokines (IL-6, IL-8 and IL-1β), endothelial dysfunction, and increased blood coagulability [6,7]. These not only affect fetal growth but also reduce birth weight [8].

Although developing countries have made efforts to improve the health and quality of life of their inhabitants, they have faced the urgent need to increase their domestic industrial production to improve economic growth. Increased industrialization directly affects the environment, specifically air quality, and produces high pollution levels equivalent to those in industrialized countries [9].

Studies relating air pollution to neonatal characteristics are few or nonexistent in Latin America and Chile. Therefore, this study was designed to determine the association of prematurity and low birth weight with gestational exposure to PM_2.5_ and PM_10_ particulate matter in Chileans newborns.

## 2. Materials and Methods

### 2.1. Study Design

An analytical cross-sectional study design was used.

### 2.2. Samples

The sample consisted of 595,369 Chilean newborns who were born between 2014 and 2016 in the north, central, south, and Patagonian zones of the country and included in the records of the Department of Health Statistics and Information (DEIS) of the Chilean Ministry of Health. We excluded newborns from multiple births and those with less than 24 weeks of gestation.

The following categories were included in the analysis: sex (male/female), delivery (term ≥ 37 weeks or preterm < 37 weeks), birth weight (normal ≥ 2500 g or low < 2500 g), and living status (urban/rural). The neonatal nutritional evaluation was performed using the standardized references established for Chile [10].

Ethical review and approval were waived for this study due to we used public database.

### 2.3. Air Pollution

Air quality records (PM_2.5_ and PM_10_) were obtained from the Air Quality Information System (SINCA) of the Chilean Ministry of the Environment (https://sinca.mma.cl, accessed on 14 January 2020). PM_10_ and PM_2.5_ records were obtained from eight national monitoring stations (determined by gravimetric analysis). Exposure was determined by the mean amount of pollutant (PM_2.5_, PM_10_) for each gestational trimester, which were calculated from the date of delivery.

### 2.4. Statistical Analysis

A binary multiple logistic regression model was performed to evaluate the association of prematurity and low birth weight (LBW) with air pollutants. Results were expressed as raw and adjusted odds ratio (OR) and confidence intervals (95% CI). The adjusted model included the maternal characteristics of age, living, educational level, and type of delivery. The statistical analysis was performed with STATA 15.0 software (StataCorp LLC, College Station, TX, USA).

## 3. Results

Table 1 provides the main sociodemographic and anthropometric characteristics of the study subjects. The majority of Chilean newborns were female, of urban living, with term gestational age, and adequate birth weight for gestational age.

In addition, maternal age was 28.1 ± 6.5 years, gestation was 38.4 ± 1.8 weeks, and weight and length at birth of 3310.8 ± 545.7 g and 49.1 ± 2.7 cm, respectively. It was established that mean PM_2.5_ and PM_10_ concentrations were 25.5 µg/m^3^ and 55.3 µg/m^3^, respectively. No concentration differences were found according to the gestational trimester (data not shown).

The logistic regression model showed that adjusting for the newborn’s sex, maternal age and living, PM_2.5_ increased the risk of LBW (OR: 1.031; 95% CI: 1.004–1.059) when exposure occurred in the second trimester, while PM_10_ affected it in all gestational trimesters. It was also found that PM_10_ exposure in any gestational trimester increased the risk of prematurity (Table 2).

## 4. Discussion

Preterm and/or LBW newborns face physiological problems in adapting to the new environment since they do not have the same physiological adaptation and development opportunities as a term newborn [11].

The prevalence of prematurity in our study was 7.4%; these results concur with those re-ported by López-Orellana [12] who indicate a sustained increase of prematurity in Chile from 1990 (4.7%) to 2012 (6.0%). Increased prematurity in Chile is not an isolated case given that it has also been reported in Western countries.

We found that the annual mean for PM_2.5_ and PM_10_ was 25.5 µg/m^3^ and 55.3 µg/m^3^, respectively. Higher means were observed in the Patagonian zone with values of 55.7 µg/m^3^ for PM_2.5_ and 69.9 µg/m^3^ for PM_10_. The obtained values are 3–5 times higher than those established by the World Health Organization (WHO), which specifies maximum acceptable annual means of 5.0 µg/m^3^ for PM_2.5_ and 15.0 µg/m^3^ for PM_10_ [13]. Our findings are slightly higher than the maximum levels allowed according to Chilean legislation for MP 20.0 µg/m^3^ (PM_2.5_) and 50.0 µg/m^3^ (PM_10_) annual concentration [14,15].

According to our findings, gestational exposure to PM_2.5_ and PM_10_ increases the risk of LBW. However, weight was affected by PM_2.5_ only when exposure occurred in the second gestational trimester. In their meta-analysis, Zhu et al. [16] show that there is a positive association between PM_2.5_ and LBW (OR: 1.05; 95% CI: 1.02–1.07), and their values are also similar (OR: 1.09; 95% CI: 1.03–1.15) to those reported by Sun et al. [17]. As for the association between PM_10_ and LBW, Liu et al. [18] found that it was significant only when the complete gestational period is evaluated (OR: 1.01; 95% CI: 1.01–1.04).

When evaluating the association between prematurity and exposure to particulate matter, we found a positive association for PM_10_ with prematurity in all gestational trimesters. The study by Li et al. [19] with a population of 1.3 million newborns, showed that PM_10_ exposure in the first (OR: 1.02; 95% CI: 1.02–1.02) and second trimester (OR: 1.01; 95% CI: 1.01–1.02) was associated with the risk of prematurity, but not so with exposure during the third trimester (OR: 0.99; 95% CI: 0.99–1.00). However, no association between PM_2.5_ and prematurity in any trimester of exposure was found in our study. In other studies of populations exposed to annual pollution means similar to those reported in our study, results are contradictory [20].

Chronic exposure to particulate matter could affect inflammation markers, gene expression modification, and reproductive, placental, and fetal health alteration [1,5,11,21]. During gestation, methylation of the IGF2/h19 group appears to be a potential mechanism underlying the association between exposure to particulate matter and fetal growth [22].

The cost attributable to preterm deliveries caused by particulate matter could exceed 5.090 million USD [23]. It is therefore imperative to continue studying the effect of environmental pollution on neonatal health in order to generate enough evidence to ensure health care and sustainable economic growth.

Some limitations of our study should be noted. Given the nature of the database, no information related to maternal morbidity was available and some women may reside in a different location than where they work. This information could have contributed to a more in-depth analysis of our findings.

## 5. Conclusions

An increase in the prevalence of preterm newborns has been observed in Chile, which is currently 7.4%. Chilean pregnant are exposed to annual means for PM_2.5_ and PM_10_, three to five times higher than the maximum acceptable as recommended by the WHO. The PM_10_ particulate matter was associated with both prematurity and low birth weight in all of the trimesters of exposure. The PM_2.5_ particulate matter was only associated with low birth weight when exposure occurred in the second gestational trimester.

## Figures and Tables

**Table 1:** Sociodemographic and anthropometric characteristics of Chilean newborns.

Variable	*n* = 595,369	%
Sex		
Female	303,149	50.9
Male	292,220	49.1
Gestational age		
Term	551,220	92.6
Preterm	44,149	7.4
Birth weight		
Normal	563,139	86.5
Low birth weight	32,230	5.5
Weight for gestational age		
Adequate	465,762	78.2
Not adequate	129,607	21.8
Living		
Urban	548,958	92.2
Rural	46,411	7.8

**Table 2:** Multiple logistic regression model for the association of prematurity and low birth weight with air pollutants.

Variables	Pollutant	Trimester	OR _raw_ (CI)	OR _ajusted_ (CI)
Low birth weight	PM_2.5_	1	0.990 (0.974–1.025)	1.050 (0.979–1.032)
		2	1.039 (1.012–1.066)	1.031 (1.004–1.059)
		3	1.028 (1.002–1.055)	1.017 (0.990–1.044)
	PM_10_	1	1.096 (1.069–1.123)	1.080 (1.053–1.109)
		2	1.156 (1.127–1.185)	1.144 (1.114–1.175)
		3	1.140 (1.112–1.169)	1.131 (1.102–1.162)
Prematurity	PM_2.5_	1	1.000 (0.978–1.022)	0.999 (0.978–1.021)
		2	1.024 (1.001–1.046)	1.020 (0.998–1.043)
		3	1.010 (0.988–1.033)	1.006 (0.984–1.029)
	PM_10_	1	1.056 (1.034–1.079)	1.050 (1.028–1.073)
		2	1.091 (1.067–1.115)	1.083 (1.059–1.107)
		3	1.091 (1.067–1.115)	1.081 (1.058–1.105)

Adjusted for sex, maternal age, and maternal living.

## Data Availability

Please check www.minsal.cl (for Chilean newborns data) (accessed on 15 October 2019) and https://sinca.mma.cl (for air quality records) (accessed on 14 January 2020).
